# SOCS3 Promoter Hypermethylation Is a Favorable Prognosticator and a Novel Indicator for G-CIMP-Positive GBM Patients

**DOI:** 10.1371/journal.pone.0091829

**Published:** 2014-03-14

**Authors:** Ying Feng, Zheng Wang, Zhaoshi Bao, Wei Yan, Gan You, Yinyan Wang, Huimin Hu, Wei Zhang, Quangeng Zhang, Tao Jiang

**Affiliations:** 1 Department of Immunology, Institute of Basic Medical Sciences, Capital Medical University, Beijing, China; 2 Department of Neurosurgery, Beijing Tiantan Hospital, Capital Medical University, Beijing, China; 3 Beijing Neurosurgical Institute, Beijing, China; University of Navarra, Spain

## Abstract

**Background:**

Hypermethylation of the suppressor of cytokine signaling 3(SOCS3) promoter has been reported to predict a poor prognosis in several cancers including glioblstoma multiforme (GBM). We explored the function of SOCS3 promoter hypermethylation in GBM cohorts, including analysis of the CpG island methylator phenotype (CIMP), when a large number of gene loci are simultaneously hypermethylated.

**Methods:**

A whole genome promoter methylation profile was performed in a cohort of 33 GBM samples, with 13 long-term survivors (LTS; overall survival ≥ 18 months) and 20 short-term survivors (STS; overall survival ≤ 9 months). The SOCS3 promoter methylation status was compared between the two groups. In addition, we investigated the relationship of SOCS3 promoter methylation and G-CIMP status.

**Results:**

Interestingly, in our present study, we found that SOCS3 promoter methylation was statistically significantly higher in the 13 LTS than that in the 20 STS. Furthermore, high SOCS3 promoter methylation detected via pyro-sequencing predicted a better prognosis in an independent cohort containing 62 GBM patients. This correlation was validated by the dataset from the Cancer Genome Atlas(TCGA) and the Chinese Cancer Genome Atlas(CGGA). In addition, we found that hypermethylation of the SOCS3 promoter was tightly associated with the G-CIMP-positive GBM patients.

**Conclusions:**

Using a total of 359 clinical samples, we demonstrate that SOCS3 promoter hypermethylation status has a favorable prognostic value in GBM patients because of whole genome methylation status. Particularly, the hypermethylation of the SOCS3 promoter indicates positive G-CIMP status.

## Introduction

Glioblastoma is the most malignant primary brain tumor in adults with an overall survival rate of about 1.5 years even when treated with radical regimens including surgical resection, and radiotherapy with concomitant and/or adjuvant temozolomide chemotherapy[1]. Although the exact mechanism of GBM development and progression is still unknown, certain molecular biomarkers are related to tumorigenesis and progression of GBM at the genetic, epigenetic, and transcriptional levels[2], [3], [4], [5].

However, markers for GBM that have prognostic value in signaling transduction pathways have not been fully elucidated yet.The Janus kinase/signal transducer and activator of transcription 3 (JAK/STAT3) signaling pathways transmits extracellular signals into the nucleus where it regulates DNA transcription and activity in the cell[6]. The suppressor of cytokine signaling 3 (SOCS3) is an endogenous inhibitor of the JAK/STAT3 signaling pathway, modulating cell activities via suppressing transcription. Recently, some studies have reported that SOCS3 functions as a tumor suppressor in multiple tumor types, including GBM [7], [8], [9], [10].

DNA methylation is a precisely regulated process in normal cells that becomes drastically modified in cancer cells[5], [11], [12]. Hypomethylation of oncogene promoters and hypermethylation of tumor suppressor gene promoters are pivotal alterations in cancer development[13], [14]. Moreover, DNA methylation is typically a stable and inheritable epigenetic pattern that can persist for several cell generations, which potentially broadens its clinical practical applicability[15].

Hypermethylation of oncogenic genes is a favorable indictor for GBM patients. A variety of studies have reported that hypermethylation of the SOCS3 promoter predicts poor prognosis in certain cancers, including GBM[16], [17], [18], [19]. However, in our study, hypermethylation of the SOCS3 promoter was associated with better outcomes for GBM patients. In addition, we found that hypermethylation of the SOCS3 promoter in GBM was tightly associated with the G-CIMP-positive GBM patients.

## Materials and Methods

### Patients and samples

All patients with primary GBM were from the Chinese Glioma Genome Atlas (CGGA) who underwent surgical resection between January 2006 and December 2010 and subsequently received radiotherapy and/or adjuvant temozolomide. Tumor tissue samples were obtained by surgical resection before the treatment with radiation and/or chemotherapy. Specimens were snap-frozen in liquid nitrogen until nucleic acid extraction. We invited two independent neuropathologists to evaluate the specimens histologically. Primary and secondary glioblastoma were distinguished based on patients' clinical history record. Written informed consents were obtained from the patients (or their families). No minors/children patients were included in our research. This study was approved by the Ethics Committee of Capital Medical University, Beijing, China.

### DNA extraction

A hematoxylin and eosin-stained frozen section was prepared for assessment of the percentage of tumor cells before DNA extraction. Only samples with greater than 80% tumor cells were selected. Genomic DNA was isolated from frozen tumor tissues using the QIAamp DNA Mini Kit (Qiagen) according to the manufacturer's protocol. DNA concentration and quality were measured using the NanoDrop ND-1000 spectrophotometer (NanoDrop Technologies, Houston, TX).

### Genome-wide DNA methylation profiling

We used the Illumina Infinium HumanMethylation27 Bead-Chip (Illumina Inc.) [20]. The BeadChip contains 27,578 highly informative CpG sites covering more than 14,000 human RefSeq genes, and allows researchers to investigate all of these sites per sample at a single nucleotide resolution. Bisulfite modification of DNA, chip processing and data analysis were performed following the manufacturer's manual at the Wellcome Trust Centre for Human Genetics Genomics Lab in Oxford, UK. The array results were analyzed with the BeadStudio software (Illumina). We have deposited our dataset on Gene Expression Omnibus (GEO) and the GEO accession number is GSE53228.

### Pyrosequencing analysis of SOCS3

Pyrosequencing was supported by Genetech (Shanghai, China) and performed using the PyroMark Q96 ID System (Qiagen) according to the manufacturer's protocol. Bisulfite modification of the DNA was accomplished using the EpiTect Kit (Qiagen). The beta value is a quantitative measure of DNA methylation levels of specific CpGs using the ratio of intensities between methylated and unmethylated alleles[21].

### Statistical analysis

T-tests were performed using GraphPad Prism 5. Kaplan–Meier survival curves were obtained, and differences in the overall survival were tested for statistical significance using the log-rank test (GraphPad Prism 5). P<0.05 was considered significant.

## Results

### Hypermethylation of the SOCS3 promoter predicts better prognosis for GBM patients

**Figure 1:**
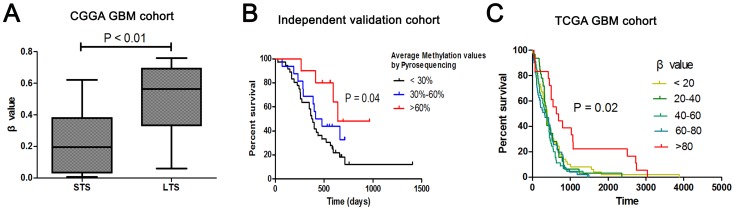
SOCS3 methylation status in STS and LTS group and validiation cohort. **A.** In the CGGA GBM cohort, the β values of the SOCS3 promoter of the two groups (STS group and LTS group) are significantly different (P<0.01). **B.** In an independent validation cohort, survival analysis showed that three groups divided by average methylation values are significantly different (P<0.04). **C.** In the TCGA GBM cohort, the group with β value>80 percent (red) has a significantly longer survival than the other four groups (P = 0.02).

Our test cohort consisted of 13 long-term survivors (LTS) whose overall survivals were more than 18 months, and 20 short-term survivors (STS) whose overall survivals were less than 9 months. The β values of the SOCS3 promoter of the two groups are shown in Figure 1A, which displayed statistically significant difference (P<0.01) between the STS group and the LTS group. Thus we inferred that hypermethylation of the SOCS3 promoter may correlate with favorable prognosis in GBM patients. Our results were validated in an independent cohort containing 62 GBM samples from Tiantan Hospital, Beijing, China. According to average methylation values measured by pyrosequencing (Figure S1), 62 samples of the independent validation cohort were divided into three groups (Figure 1B): average methylation values <30%, average methylation values between 30% to 60%, and average methylation values>60%. A comparison of the three groups demonstrated that there was a statistically significant difference in the patients' survival (P = 0.04) among the groups. Our findings were validated using the Cancer Genome Atlas (TCGA) dataset (n = 264). In the TCGA validating cohort, we divided all the samples into five groups according to their β values. A Kaplan-Meier curve of the survival of these 264 patients is shown in Figure 1C. Significant difference was found between the groups with a β value>80% and the groups with relatively lower β values.

### Hypermethylation of the SOCS3 promoter is associated with G-CIMP-positive GBM patients

**Figure 2:**
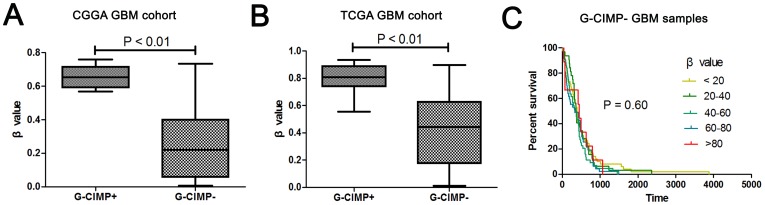
G-CIMP status in CGGA and TCGA cohort. **A.** In the CGGA GBM cohort, the β values of the SOCS3 promoter of the two groups (G-CIMP-positive group and G-CIMP-negative group) displayed statistically significant difference (P<0.01). **B.** In the TGGA GBM cohort, the β values of the SOCS3 promoter of the two groups (G-CIMP-positive group and G-CIMP-negative group) also displayed statistically significant difference (P<0.01). **C.** In the G-CIMP-negative TCGA samples, there was no significant difference among the five groups (P = 0.60).

In addition, we found that hypermethylation of the SOCS3 promoter is tightly associated with G-CIMP-positive GBM patients in two independent cohorts, the CGGA GBM and the TCGA GBM cohorts. In the CGGA cohort, the β value of the G-CIMP-positive group was 0.66, which is significantly higher than 0.26, the β value of the G-CIMP-negative group (Figure 2A, P<0.01). Similarly, statistically significant difference was observed in the G-CIMP-positive group compared with the G-CIMP-negative group in the TCGA GBM cohort with β values of 0.81 and 0.41, respectively (Figure 2B, P<0.01). In another TCGA cohort of which all the 242 GBM samples were G-CIMP-negative, we divided the samples into five groups according the average β values, similar to the TCGA validating cohort (Figure 1C). Our analysis demonstrated that the β value provided little clinical prognostic value for these patients as shown in the Kaplan-Meier curve (Figure 2C, P = 0.6). These results indicate that the prognostic value of hypermethylation of the SOCS3 promoter was tightly associated only with G-CIMP-positive GBM samples.

### A novel indicator for G-CIMP-positive GBM patients

**Figure 3:**
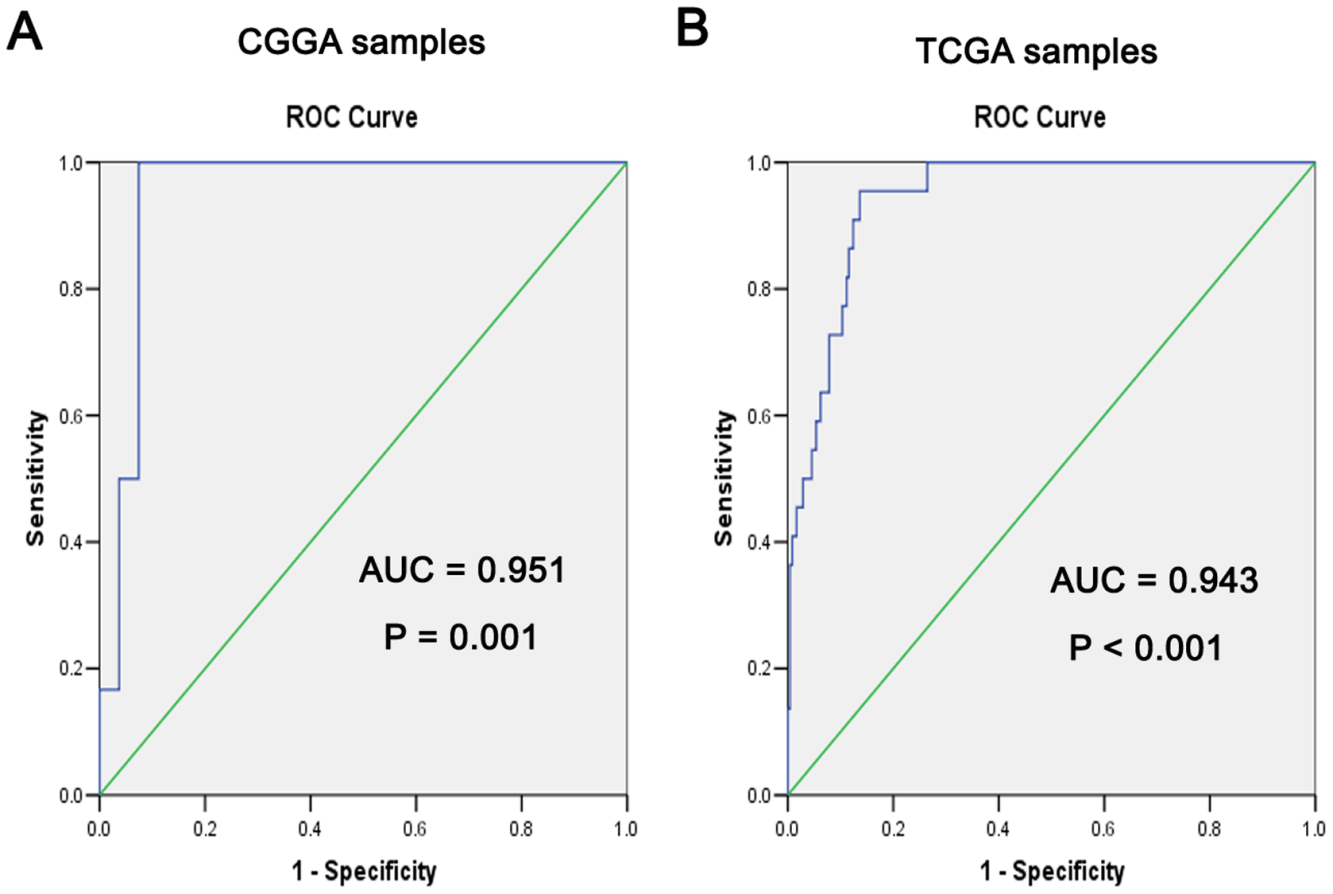
Hypermethylation of SOCS3 promoter is consistent with G-CIMP. **A.** In the CGGA samples, a ROC curve demonstrated a tight relationship between hypermethylation of the SOCS3 promoter and G-CIMP-positive status (AUC = 0.951, P = 0.001). **B.** In the TCGA samples, a similar relationship between hypermethylation of the SOCS3 promoter and G-CIMP-positive status is shown in the ROC curve (AUC = 0.943, P<0.001).

We performed a receiver operating characteristic curve (ROC curve) between the hypermethylation of the SOCS3 promoter and G-CIMP to define the exact relationship. According to our data analysis, statistical significance was observed in CGGA samples (AUC = 0.951, P = 0.001) (Figure 3A). These results indicate a robust relationship between the hypermethylation of the SOCS3 promoter and G-CIMP positive. Thus, hypermethylation of the SOCS3 promoter is a de novo indicator for G-CIMP. To validate our results, TCGA samples were subsequently used to verify this relationship; the results are even better than that of CGGA samples (AUC = 0.943, P<0.001) (Figure 3B).

## Discussion

Glioblastoma is the most malignant primary brain tumor in adults, with insidious development, rapid progression and poor outcomes. Alterations in cell signaling pathways may be associated with the development and progression of GBM. Some prognostic bio-markers involved in signaling pathways have been identified. Hypermethylation of the SOCS3 promoter has been associated with a poor outcome for GBM. From our present research, we draw an opposite conclusion to previous studies and show that hypermethylation of the SOCS3 promoter predicts an improved prognosis for GBM patients.

DNA methylation is a common regulatory process which influences cell activities including transcription in normal cells. DNA methylation frequently becomes drastically aberrantly altered in cancer cells[12]. Hypomethylation of oncogene promoters and hypermethylation of tumor suppressor gene promoters are pivotal alterations in cancer development.

The CpG island methylator phenotype (CIMP) is a methylation status when a large number of gene loci are simultaneously hypermethylated, probably as consequence of mutations of methyltransferases or histone-modifying proteins[22], aging[22], virus exposure[23], [24], chronic inflammation[25], [26] or other underlying factors. Reportedly, CIMP was observed in many tumors, including colorectal cancer[27], [28], adrenocortical carcinomas[29], gastric tumors[30], [31], liver cancer[23], esophagus cancer[32], ovarian cancers[33] and acute myelogenous leukemia[34], [35]. In different tumors, CIMP of the whole tumor genome affects different specific genes and functions differently, either as favorable or unfavorable predictors for patients. Poorer outcome was observed in patients who suffered adrenocortical carcinomas with the existence of CIMP[29]. Nevertheless, according to previous research, in gastric carcinoma, the prognosis of the patients without CIMP was significantly worse compared with that of patients with CIMP[29]. Such evidence confirms the fact that hypermethylation of the whole cancer genome does not necessarily mean better or worse outcomes for patients. Instead, it is the specific genes that are aberrantly methylated that determine outcomes[27].

G-CIMP is enriched in a subgroup of glioma, the proneural subgroup, according to the TCGA classification scheme for glioma[36]. In G-CIMP-positive samples, a large number of CpG island loci located in specific gene promoters are hypermethylated and patients usually have better outcomes[27]. According to our research and data analysis, hypermethylation of the SOCS3 promoter is highly associated with G-CIMP-positive samples and predicts improved outcomes for patients, but is not a predictor for G-CIMP-negative patients. Therefore, we conclude that SOCS3 hypermethylation status has favorable prognostic value in GBM patients because of its whole genome methylation status.

SOCS3 functions as a tumor suppressor in many cancers including GBM. According to the bio-effects of the genetic hypermethylation process, hypermethylation of tumor suppressor gene promoters theoretically is aversive for tumorigenesis or progression. Furthermore, many studies have confirmed the effect of SOCS3 in GBM samples. In G-CIMP-positive samples, as our data showed above, the SOCS3 promoter is hypermethylated along with a variety of other loci. The hypermethylation of the SOCS3 promoter is just a part of the whole genome methylation status and its negative effect on tumorigenesis or progression may be neutralized by the comprehensive genome hypermethylation[37]. This hypothesis may explain why hypermethylation of the SOCS3 promoter predicts favorable prognosis in GBM patients. In addition, other potential signaling pathways may be uncovered for which hypermethylation of the SOCS3 promoter serves as a better prognosticator. Because this single gene alteration accompanies whole genome hypermethylation, SOCS3 can be regarded as a pivotal gene that functions as a predictor for the whole genome methylation status (G-CIMP). As we revealed in this research, SOCS3 hypermethylation is a de novo indicator for G-CIMP and predicts better patients' outcomes. The prognostic value of SOCS3 hypermethylation is also practical as it is easy to perform in clinical practice and could be helpful in determining therapeutic regimens for GBM patients.

## Conclusions

In summary, we found that hypermethylation of the SOCS3 promoter predicts favorable prognosis. Our results were validated in an independent cohort containing 62 GBM samples as well as in a TCGA GBM cohort. Further investigation is needed to uncover the exact mechanism of how hypermethylation of SOCS3 promoter affects the normal processes in the cell and its relationship to tumorigenesis and progression. We also found that SOCS3 is a de novo indicator for G-CIMP-positive GBM patients.

## Supporting Information

Figure S1 **Pyrosequencing for SOCS3 promoter methylation.** This figure shows unmethylated and methylated SOCS3 promoters using pyrosequencing.
(TIF)

## References

[1] Yang P, Wang Y, Peng X, You G, Zhang W, et al.. (2013) Management and survival rates in patients with glioma in China (2004–2010): a retrospective study from a single-institution. J Neurooncol. 10.1007/s11060-013-1103-9 23483435

[2] Yan H, Parsons DW, Jin G, McLendon R, Rasheed BA, et al (2009) IDH1 and IDH2 mutations in gliomas. N Engl J Med 360: 765–773. 19228619 10.1056/NEJMoa0808710 PMC2820383

[3] Zhang W, Zhang J, Hoadley K, Kushwaha D, Ramakrishnan V, et al (2012) miR-181d: a predictive glioblastoma biomarker that downregulates MGMT expression. Neuro Oncol 14: 712–719. 22570426 10.1093/neuonc/nos089 PMC3367855

[4] Singh D, Chan JM, Zoppoli P, Niola F, Sullivan R, et al (2012) Transforming fusions of FGFR and TACC genes in human glioblastoma. Science 337: 1231–1235. 22837387 10.1126/science.1220834 PMC3677224

[5] Zhang W, Zhang J, Yan W, You G, Bao Z, et al (2013) Whole-genome microRNA expression profiling identifies a 5-microRNA signature as a prognostic biomarker in Chinese patients with primary glioblastoma multiforme. Cancer 119: 814–824. 22990979 10.1002/cncr.27826

[6] Aaronson DS, Horvath CM (2002) A road map for those who don't know JAK-STAT. Science 296: 1653–1655. 12040185 10.1126/science.1071545

[7] Martini M, Pallini R, Luongo G, Cenci T, Lucantoni C, et al (2008) Prognostic relevance of SOCS3 hypermethylation in patients with glioblastoma multiforme. Int J Cancer 123: 2955–2960. 18770864 10.1002/ijc.23805

[8] Lindemann C, Hackmann O, Delic S, Schmidt N, Reifenberger G, et al (2011) SOCS3 promoter methylation is mutually exclusive to EGFR amplification in gliomas and promotes glioma cell invasion through STAT3 and FAK activation. Acta Neuropathol 122: 241–251. 21590492 10.1007/s00401-011-0832-0

[9] Li Y, Deuring J, Peppelenbosch MP, Kuipers EJ, de Haar C, et al (2012) IL-6-induced DNMT1 activity mediates SOCS3 promoter hypermethylation in ulcerative colitis-related colorectal cancer. Carcinogenesis 33: 1889–1896. 22739025 10.1093/carcin/bgs214

[10] Isomoto H (2009) Epigenetic alterations in cholangiocarcinoma-sustained IL-6/STAT3 signaling in cholangio- carcinoma due to SOCS3 epigenetic silencing. Digestion 79 Suppl 12–8. 19153483 10.1159/000167859

[11] Rivera AL, Pelloski CE, Gilbert MR, Colman H, De La Cruz C, et al (2010) MGMT promoter methylation is predictive of response to radiotherapy and prognostic in the absence of adjuvant alkylating chemotherapy for glioblastoma. Neuro Oncol 12: 116–121. 20150378 10.1093/neuonc/nop020 PMC2940581

[12] Laffaire J, Everhard S, Idbaih A, Criniere E, Marie Y, et al (2011) Methylation profiling identifies 2 groups of gliomas according to their tumorigenesis. Neuro Oncol 13: 84–98. 20926426 10.1093/neuonc/noq110 PMC3018904

[13] Wolff EM, Byun HM, Han HF, Sharma S, Nichols PW, et al (2010) Hypomethylation of a LINE-1 promoter activates an alternate transcript of the MET oncogene in bladders with cancer. PLoS Genet 6: e1000917. 20421991 10.1371/journal.pgen.1000917 PMC2858672

[14] Kloten V, Becker B, Winner K, Schrauder MG, Fasching PA, et al (2013) Promoter hypermethylation of the tumor-suppressor genes ITIH5, DKK3, and RASSF1A as novel biomarkers for blood-based breast cancer screening. Breast Cancer Res 15: R4. 23320751 10.1186/bcr3375 PMC3672828

[15] Gaspar-Maia A, Alajem A, Meshorer E, Ramalho-Santos M (2011) Open chromatin in pluripotency and reprogramming. Nat Rev Mol Cell Biol 12: 36–47. 21179060 10.1038/nrm3036 PMC3891572

[16] Fourouclas N, Li J, Gilby DC, Campbell PJ, Beer PA, et al (2008) Methylation of the suppressor of cytokine signaling 3 gene (SOCS3) in myeloproliferative disorders. Haematologica 93: 1635–1644. 18815196 10.3324/haematol.13043

[17] Sutherland KD, Lindeman GJ, Choong DY, Wittlin S, Brentzell L, et al (2004) Differential hypermethylation of SOCS genes in ovarian and breast carcinomas. Oncogene 23: 7726–7733. 15361843 10.1038/sj.onc.1207787

[18] Weber A, Hengge UR, Bardenheuer W, Tischoff I, Sommerer F, et al (2005) SOCS-3 is frequently methylated in head and neck squamous cell carcinoma and its precursor lesions and causes growth inhibition. Oncogene 24: 6699–6708. 16007169 10.1038/sj.onc.1208818

[19] He B, You L, Uematsu K, Zang K, Xu Z, et al (2003) SOCS-3 is frequently silenced by hypermethylation and suppresses cell growth in human lung cancer. Proc Natl Acad Sci U S A 100: 14133–14138. 14617776 10.1073/pnas.2232790100 PMC283558

[20] Hill VK, Ricketts C, Bieche I, Vacher S, Gentle D, et al (2011) Genome-wide DNA methylation profiling of CpG islands in breast cancer identifies novel genes associated with tumorigenicity. Cancer Res 71: 2988–2999. 21363912 10.1158/0008-5472.CAN-10-4026

[21] Kuan PF, Wang S, Zhou X, Chu H (2010) A statistical framework for Illumina DNA methylation arrays. Bioinformatics 26: 2849–2855. 20880956 10.1093/bioinformatics/btq553 PMC3025715

[22] Issa JP (2004) CpG island methylator phenotype in cancer. Nat Rev Cancer 4: 988–993. 15573120 10.1038/nrc1507

[23] Shen L, Ahuja N, Shen Y, Habib NA, Toyota M, et al (2002) DNA methylation and environmental exposures in human hepatocellular carcinoma. J Natl Cancer Inst 94: 755–761. 12011226 10.1093/jnci/94.10.755

[24] Kang GH, Lee S, Kim WH, Lee HW, Kim JC, et al (2002) Epstein-barr virus-positive gastric carcinoma demonstrates frequent aberrant methylation of multiple genes and constitutes CpG island methylator phenotype-positive gastric carcinoma. Am J Pathol 160: 787–794. 11891177 10.1016/S0002-9440(10)64901-2 PMC1867170

[25] Issa JP, Ahuja N, Toyota M, Bronner MP, Brentnall TA (2001) Accelerated age-related CpG island methylation in ulcerative colitis. Cancer Res 61: 3573–3577. 11325821

[26] Zochbauer-Muller S, Fong KM, Virmani AK, Geradts J, Gazdar AF, et al (2001) Aberrant promoter methylation of multiple genes in non-small cell lung cancers. Cancer Res 61: 249–255. 11196170

[27] Weisenberger DJ, Siegmund KD, Campan M, Young J, Long TI, et al (2006) CpG island methylator phenotype underlies sporadic microsatellite instability and is tightly associated with BRAF mutation in colorectal cancer. Nat Genet 38: 787–793. 16804544 10.1038/ng1834

[28] Goel A, Nagasaka T, Arnold CN, Inoue T, Hamilton C, et al (2007) The CpG island methylator phenotype and chromosomal instability are inversely correlated in sporadic colorectal cancer. Gastroenterology 132: 127–138. 17087942 10.1053/j.gastro.2006.09.018

[29] Barreau O, Assie G, Wilmot-Roussel H, Ragazzon B, Baudry C, et al (2013) Identification of a CpG island methylator phenotype in adrenocortical carcinomas. J Clin Endocrinol Metab 98: E174–184. 23093492 10.1210/jc.2012-2993

[30] Oue N, Mitani Y, Motoshita J, Matsumura S, Yoshida K, et al (2006) Accumulation of DNA methylation is associated with tumor stage in gastric cancer. Cancer 106: 1250–1259. 16475210 10.1002/cncr.21754

[31] Kusano M, Toyota M, Suzuki H, Akino K, Aoki F, et al (2006) Genetic, epigenetic, and clinicopathologic features of gastric carcinomas with the CpG island methylator phenotype and an association with Epstein-Barr virus. Cancer 106: 1467–1479. 16518809 10.1002/cncr.21789

[32] Eads CA, Lord RV, Kurumboor SK, Wickramasinghe K, Skinner ML, et al (2000) Fields of aberrant CpG island hypermethylation in Barrett's esophagus and associated adenocarcinoma. Cancer Res 60: 5021–5026. 11016622

[33] Strathdee G, Appleton K, Illand M, Millan DW, Sargent J, et al (2001) Primary ovarian carcinomas display multiple methylator phenotypes involving known tumor suppressor genes. Am J Pathol 158: 1121–1127. 11238060 10.1016/S0002-9440(10)64059-X PMC1850352

[34] Claus R, Plass C, Armstrong SA, Bullinger L (2010) DNA methylation profiling in acute myeloid leukemia: from recent technological advances to biological and clinical insights. Future Oncol 6: 1415–1431. 20919827 10.2217/fon.10.110

[35] Toyota M, Kopecky KJ, Toyota MO, Jair KW, Willman CL, et al (2001) Methylation profiling in acute myeloid leukemia. Blood 97: 2823–2829. 11313277 10.1182/blood.v97.9.2823

[36] Noushmehr H, Weisenberger DJ, Diefes K, Phillips HS, Pujara K, et al (2010) Identification of a CpG island methylator phenotype that defines a distinct subgroup of glioma. Cancer Cell 17: 510–522. 20399149 10.1016/j.ccr.2010.03.017 PMC2872684

[37] You JS, Jones PA (2012) Cancer genetics and epigenetics: two sides of the same coin? Cancer Cell 22: 9–20. 22789535 10.1016/j.ccr.2012.06.008 PMC3396881

