# Supplementary figures and images for: SOCS3 Promoter Hypermethylation Is a Favorable Prognosticator and a Novel Indicator for G-CIMP-Positive GBM Patients
Source: PLoS One. 2014 Mar 14;9(3):e91829. doi: 10.1371/journal.pone.0091829 (PMC3954800; doi:10.1371/journal.pone.0091829)

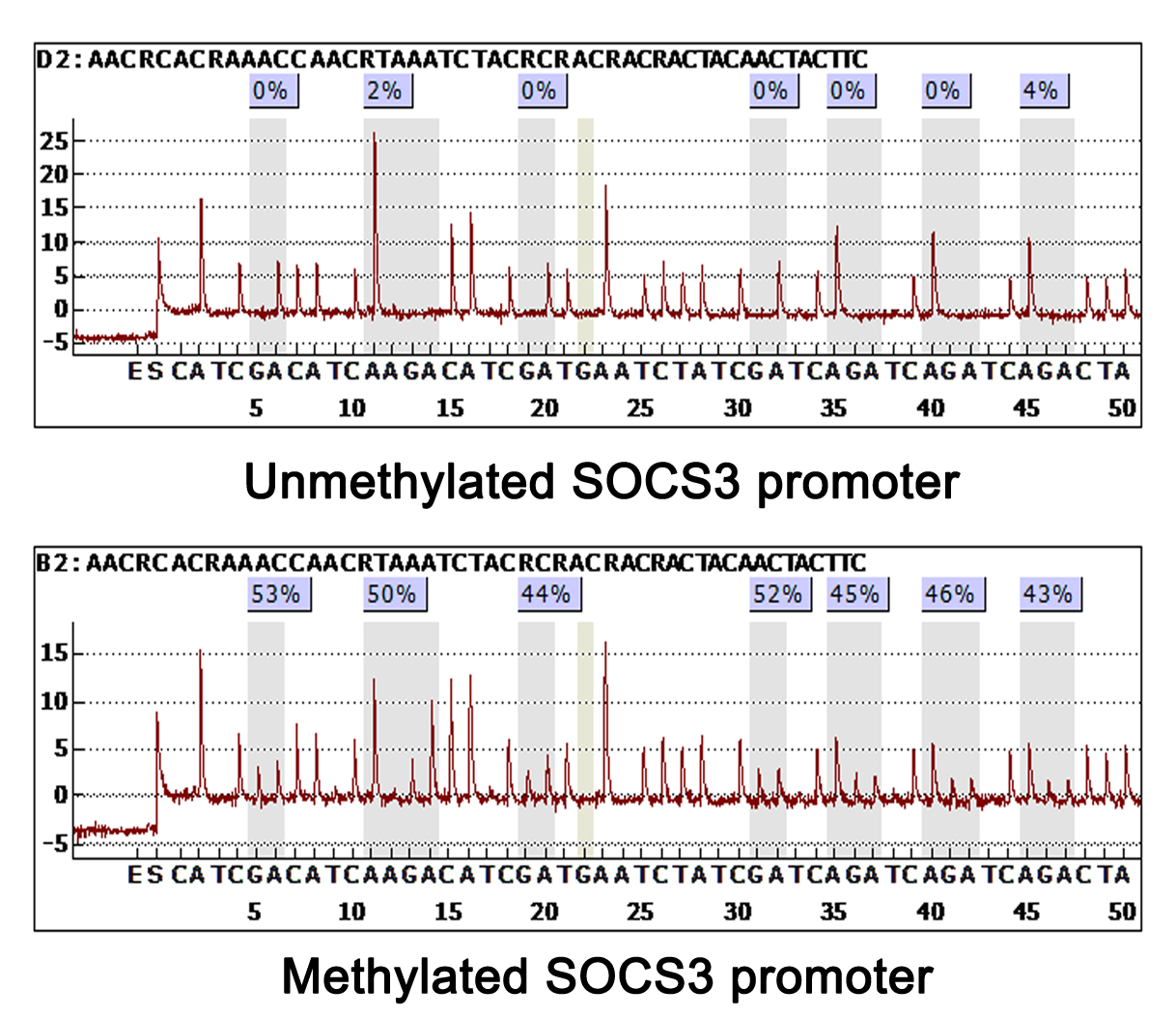

Supplement: Figure S1 — Pyrosequencing for SOCS3 promoter methylation. This figure shows unmethylated and methylated SOCS3 promoters using pyrosequencing. (TIF) [file pone.0091829.s001.tif]
